# The neighborhood built environment and COVID-19 hospitalizations

**DOI:** 10.1371/journal.pone.0286119

**Published:** 2023-06-14

**Authors:** Alessandro Rigolon, Jeremy Németh, Brenn Anderson-Gregson, Ana Rae Miller, Priyanka deSouza, Brian Montague, Cory Hussain, Kristine M. Erlandson, Sarah E. Rowan

**Affiliations:** 1 Department of City and Metropolitan Planning, The University of Utah, Salt Lake City, Utah, United States of America; 2 Department of Urban and Regional Planning, University of Colorado Denver, Denver, Colorado, United States of America; 3 Department of Medicine, Division of Infectious Diseases, University of Colorado Anschutz Medical Campus, Denver, Colorado, United States of America; 4 Division of Infectious Diseases, Denver Health and Hospital Authority, Denver, Colorado, United States of America; New York University Grossman School of Medicine, UNITED STATES

## Abstract

Research on the associations between the built environment and COVID-19 outcomes has mostly focused on incidence and mortality. Also, few studies on the built environment and COVID-19 have controlled for individual-level characteristics across large samples. In this study, we examine whether neighborhood built environment characteristics are associated with hospitalization in a cohort of 18,042 individuals who tested positive for SARS-CoV-2 between May and December 2020 in the Denver metropolitan area, USA. We use Poisson models with robust standard errors that control for spatial dependence and several individual-level demographic characteristics and comorbidity conditions. In multivariate models, we find that among individuals with SARS-CoV-2 infection, those living in multi-family housing units and/or in places with higher particulate matter (PM_2.5_) have a higher incident rate ratio (IRR) of hospitalization. We also find that higher walkability, higher bikeability, and lower public transit access are linked to a lower IRR of hospitalization. In multivariate models, we did not find associations between green space measures and the IRR of hospitalization. Results for non-Hispanic white and Latinx individuals highlight substantial differences: higher PM_2.5_ levels have stronger positive associations with the IRR of hospitalization for Latinx individuals, and density and overcrowding show stronger associations for non-Hispanic white individuals. Our results show that the neighborhood built environment might pose an independent risk for COVID-19 hospitalization. Our results may inform public health and urban planning initiatives to lower the risk of hospitalization linked to COVID-19 and other respiratory pathogens.

## 1. Introduction

The COVID-19 pandemic has caused the deaths of over 6 million people worldwide including more than one million deaths in the United States [1]. Initially, hospitals were overwhelmed with critically ill patients, and now, even among those who did not experience severe illness, long-term symptoms following COVID-19 have been unusually common [2,3]. Beyond direct health effects, the pandemic caused economic devastation and educational setbacks for children [4,5]. Despite the availability of vaccines, the pandemic may persist for years given the variability in vaccine uptake, viral variants, and relaxed prevention measures like masking [6]. In addressing our response to the current pandemic with the development of new variants and as we look to mitigate harm in future pandemics, it is important to understand factors that are associated with not only the risk of acquiring SARS-CoV-2 but also the risk of developing severe COVID-19 disease.

Numerous studies have demonstrated associations between increased COVID-19 morbidity and mortality and biological factors such as age, body mass index (BMI), and certain co-morbidities [7,8]. Less is known, however, about the association of non-biological risk factors, such as components of the built environment, with the COVID-19 disease burden. Although the effects of place on wealth, social mobility, and some chronic conditions like cardiovascular disease, obesity, and asthma are fairly well established [9–11], fewer studies have focused on the associations between the built environment and communicable diseases, particularly in relation to disease severity. Long-term exposure to higher levels of fine particulate matter (especially the concentration of particles having aerodynamic diameters < 2.5 μm: PM_2.5_) [12–15] and lower densities of green spaces [16–21] are predictors of COVID-19-related mortality and hospitalization risks, whereas studies focused on the impact of population density have yielded mixed results. Higher density has been associated with both higher COVID-19 rates and mortality in some studies [22–31]; others have found that higher population density was protective against SARS-CoV-2 incidence and COVID-19-related mortality [32–35].

Parsing out complicated overlapping risks such as socioeconomic status, age, comorbidities, environmental factors, and the impact of the built environment on COVID-19 disease outcomes is challenging. Most published studies of the environmental correlates of COVID-19 severity have not controlled for individual-level risk factors because they used geographies like counties or census tracts as their units of analysis (see [17,21–23,31,33,36–39]). Also, the effects of multiple different overlapping environmental exposures have not been critically evaluated in studies with a large sample, as most investigations have focused on one built environment domain at a time, such as green space (e.g., [17,36,40]) or density (e.g., [23,31,33]). Additionally, most work in this arena has looked at infection rates and mortality rather than hospitalizations, a proxy for more severe illness. Further, because people of color in the United States have experience disproportionately high rates of COVID-19 hospitalizations [41], it is important to know whether the associations between the neighborhood built environment and hospitalizations vary by race/ethnicity.

To address these gaps, we developed an extensive dataset with a variety of patient-level demographic and clinical characteristics for all persons diagnosed with SARS-CoV-2 through two large hospital systems in the Denver, Colorado, USA metropolitan area in 2020. We sought to understand 1) to *what extent the neighborhood built environment independently predicted COVID-19 hospitalizations*, *and 2) for which racial/ethnic groups the neighborhood built environment matters the most in predicting COVID-19 hospitalizations*.

## 2. Materials and methods

### 2.1. Study design and setting

We conducted an observational, retrospective analysis of all cases of SARS-CoV-2 diagnosed within the University of Colorado Health (UCH) and Denver Health (DH) healthcare systems from May 1, 2020, through December 15, 2020 (before vaccines became extensively available). We excluded cases from March and April 2020 due to limited testing for mild illness during the earliest months of the pandemic, which inflated the hospitalization rate (60% of those who tested positive were hospitalized in March 2020, as shown in our data).

UCH includes a network of hospitals and facilities throughout Colorado including the University of Colorado Hospital, a tertiary care center associated with the University of Colorado School of Medicine. DH is an integrated safety net healthcare system that includes an acute care hospital, 9 federally qualified health centers, and 17 school-based clinics [42]. UCH serves patients with a variety of payer sources while the majority of patients who receive care through DH are covered by Medicaid or uninsured. Overall, around 6.5% of those with a positive test in the UCH and DH systems were uninsured at the time of the study.

**Fig 1:**
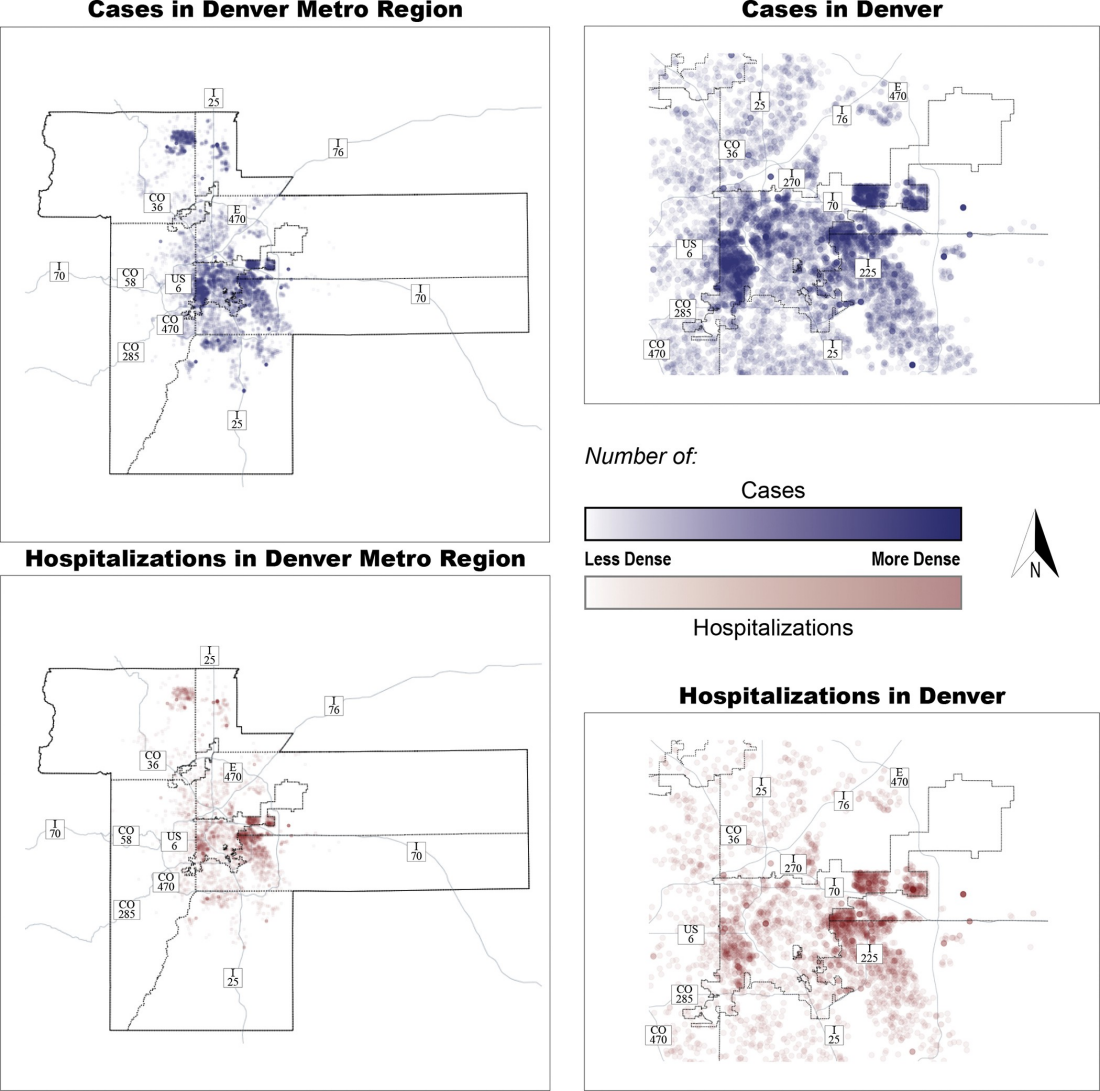
Density of SARS-CoV-2 cases and hospitalizations in the study area (Denver metropolitan area) and the City of Denver.

The analysis was limited to individuals seeking care through UCH and DH within the seven traditional counties of the Denver metropolitan area (Adams, Arapahoe, Boulder, Broomfield, Denver, Douglas, and Jefferson), plus the urbanized parts of Weld County (see Fig 1) [43]. The Denver metropolitan area is a highly urbanized region along the Colorado “Front Range” and has experienced rapid population growth over the past decade [44]. During that time, it has undergone significant population shifts characterized by densification and gentrification of the urban core and increased concentrations of lower-income people of color in some suburban communities [45].

We queried electronic health records (EHRs) from the two institutions for evidence of a positive SARS-CoV-2 polymerase chain reaction test result for individuals aged 18–100 years during the study period. Demographic variables extracted from the EHR included date of birth, race, ethnicity, gender, and home address at the time of the positive test. We excluded records with missing gender, race or ethnicity, or address (including homeless, Post Office Box only, or the address listed was for a shelter, jail, or congregate care facility such as a nursing home or rehabilitation facility). We excluded these records because environmental variables either could not be coded, as in the case of homelessness or Post Office boxes, or did not reflect the person’s interaction with the built environment, as in the case of persons in jails or congregate care facilities. We extracted health variables from disease registries and international classification of diseases (ICD) codes associated with the individual’s medical record. These included diabetes, hypertension, chronic pulmonary diseases (asthma and chronic obstructive pulmonary disease), HIV, and chronic kidney diseases. We included chronic liver disease, cardiovascular disease, pregnancy, cancer, and immunocompromised states in the query but chart reviews revealed inconsistent reporting and coding of these conditions; therefore, they were not included in the final dataset. Additional variables extracted from the EHR included current tobacco use, height, weight, and body mass index. We chose demographic variables and conditions based on current understanding of factors associated with COVID-19 disease severity [46,47].

When possible, we individually reviewed and updated records containing unlikely BMI values with additional data from chart review. We calculated missing BMI values for those patients with an available weight using the median observed height value for their gender and reported race/ethnicity. Medians heights from our study cohort were compared with national data and determined to be within 2 centimeters for all subsets [48]. We did not impute missing values for weights given the larger variability in possible weights and greater impact on BMI results. Individuals without documented BMIs or weights were thus excluded from the analyses. We calculated BMI values from the most recent height and weight data and compared these to extracted BMI values with additional chart reviews conducted in cases of discrepancies. The final BMI value assigned to each record in the dataset was the value obtained from measurements taken closest to the time of the positive SARS-CoV-2 test.

We then queried the EHRs for evidence of hospital admission at DH or UCH within two weeks of the positive SARS-CoV-2 test for individuals in the cohort. The reason for hospitalization was not routinely available, and therefore individuals could have been hospitalized for COVID-19 or unrelated reasons. In the case of multiple positive SARS-CoV-2 tests, we included only the hospitalization status associated with the initial positive test in the analysis. We did so to avoid biasing the results by including the same individual multiple times. In some cases, a patient may have been hospitalized and had multiple tests sent from different anatomic sites (e.g., nasopharyngeal, saliva) or repeated multiple times during the hospitalization due to admission requirements of long term care facilities. In other cases, individuals were discharged and readmitted in the next few weeks for unrelated issues, yet because of universal screening for SARS-CoV-2 at that point in the pandemic, still had positive results when admitted. Finally, individuals who had multiple positive tests several months apart are likely to have other predisposing factors to illness and that could also bias the results if they were included more than once.

### 2.2. Neighborhood built environment

We created a four-pronged classification of neighborhood built environment features based on our critical reading of recent reviews and theoretical articles about COVID-19 and the built environment [49–53] and our evaluation of the current empirical research on this topic. The four domains we focused on were population density and crowding (e.g., living in an apartment), environmental hazards (e.g., PM_2.5_, proximity to a highway), environmental amenities (e.g., parks access, park acreage), and mobility options (e.g., transit access, cycling infrastructure).

We recoded home addresses for individuals in the cohort to geographical coordinates using the Texas A&M University (TAMU) Geocoder (version 4.01, College Station, TX) and the Bing Maps Geocoder (version 2.0, Redmond, WA). Prior use of geocoders has been associated with the inadvertent sharing of protected health information (PHI) [54]. To ensure this did not occur, we uploaded addresses to the geocoders in small batches (less than 2,500 cases) on different days and used various internet protocol (IP) addresses to do so. Additionally, we only uploaded street addresses and unique identifiers, and we deleted addresses from the geocoders after each use. Further, the TAMU geocoder, which is the main service we used, deletes all its data every seven days [55]. If the addresses were accessed while they were temporarily stored in the geocoders, they would not be traceable to this study because the IP addresses from which the data was uploaded varied and are not clearly linked to the two hospital systems. Therefore, there would be no evidence that an individual with a given street address was associated with this study cohort.

We then used Python (version 3.6.1) to clean several variables describing the neighborhood built environment and linked them to the residential geographic coordinates using ArcGIS Pro (version 2.7, Redlands, CA). We list descriptions of these variables and their associated data sources in Table 1. We chose the specific variables for each neighborhood built environment domain based on data availability for the entire metro Denver region, alignment with variables used in previous studies on associations between the built environment and COVID-19 outcomes, and from preliminary tests to assess multicollinearity issues. We removed the population density variable due to strong multicollinearity with residential density (Pearson’s r = 0.95), and the Social Vulnerability Index (SVI) variable due to multicollinearity with percent housing burdened households (r = 0.66), percent essential workers (r = 0.67), and several other variables. In preliminary multivariate models, population density and SVI had Variance Inflation Factors above 4 [56]. For variables describing distances to specific locations (e.g., a park or a highway), we calculated the distance between home addresses and such locations. For other variables (e.g., residential density), we attributed the values of the census block groups within which the residential addresses were located (see Table 1 for more details).

**Table 1:** Independent variables describing the neighborhood built environment and neighborhood-level control variables.

Variable name	Description	Data source	Geography
*Neighborhood built environment*: *Density and crowding*			
Residential density	Households per square kilometer area	American Community Survey (ACS) Table B11012 [57]	Census block group
Percent overcrowding	Ratio of households (renter and owner occupied) with greater than 1 person per room*	ACS Table B25014 [57]	Census block group
Living in a multi-family building	Binary variable based on presence of a unit number in the provided address	UCH and DH Datasets	Individual-level
Percent multi-family units	Ratio of residential buildings that are not single units, mobile homes, boats, or RVs*	ACS Table B25024 [57]	Census block group
*Neighborhood built environment*: *Environmental hazards*			
Particulate Matter _2.5_ Level (PM_2.5_)	2016 Annual Average μg/m^3^ of PM_2.5_ (particulate matter)	2020 Environmental Protection Agency (EPA) Environmental Justice Screen [58]	Census block group
Proximity to a highway	Euclidean distance between home address and any highway in the region is less than 0.25 mile (400 m)	Colorado Department of Transportation Highway Dataset [59]	Individual-level, Euclidean distance-based
*Neighborhood built environment*: *Environmental amenities*			
Normalized Difference Vegetation Index greenness (NDVI)	Raw NDVI (Normalized Difference Vegetation Index) raster value	United States Geological Survey (USGS) eMODIS NDVI Raster [60]	Individual-level, raster value
Park access	Home address located within 0.5 miles (800 m) from the nearest park centroid; distance calculated based on street network	Primary: Denver Regional Council of Governments (DRCOG) Parks and Open Space Dataset [61]	Individual-level, network distance-based
Park acreage	Area of parks (square kilometers) intersecting with a .25 mile (400 m) network service shed around each address	Primary: DRCOG Parks and Open Space Dataset [61]	Individual-level, network distance-based
*Neighborhood built environment*: *Mobility*			
Walk Score ^®^	Each address was searched and the walk, bike, and transit scores were recorded. For addresses that did not return results, a raster was interpolated based on the surrounding data points and the missing values were assigned based on location.	Walk Score ^®^ Database [62]	Individual level
Bike Score ^®^		Walk Score ^®^ Database [62]	Individual level
Transit Score ^®^		Walk Score ^®^ Database [62]	Individual level
*Potential confounders*			
Percent housing burdened households	Ratio of households paying greater than 30% of their income on rent*	ACS Table B25074 [57]	Census block group
Percent essential workers	Industries were broken down as essential or non-essential based on the US Dept. of Homeland Security Cybersecurity & Infrastructure Security Agency (CISA) guidelines on essential jobs.*	ACS Table B08126 for the data [57]. For the definition of essential workers [63]	Census block group
Percent essential workers commuting via transit	Using the same definition of essential workers as above, ratio of essential workers commuting to work using transit*	ACS Table B08126 [57]	Census block group

Notes: All ACS data are from the 2015–2019 American Community Survey, 5-year estimates, for census block groups.

* We expressed all percent variables as ratios (e.g., sum of essential workers divided by total number of workers) and thus they range from 0 to 1. We calculated the variables for which geography is described as “Individual level” based on a person’s address (e.g., whether their address is within a 0.5 miles from a park). We calculated the variables for which geography is listed as “Census block group” based on values for the entire census block group in which the individual’s address is located (e.g., the residential density of the block group where one lives).

We also considered potential demographic neighborhood-level confounding factors that have been or could be associated with increased risk for severe COVID-19 (Table 1). These included the percentage of housing burdened households, the percentage of “essential workers,” and the percentage of “essential workers” commuting via transit in a census block group [64–67].

### 2.3. Outcome

The outcome of interest in the study was whether individuals who tested positive for SARS-CoV-2 were hospitalized. We analyzed the association between factors in the built environment around one’s home address and hospitalization in the entire cohort and in sub-cohort of individuals who identified as Hispanic/Latinx (all races) and those who identified as non-Hispanic white.

### 2.4. Statistical analysis

We used basic descriptive statistics to characterize the cohorts of SARS-CoV-2 positive patients diagnosed through UCH and DH. We calculated all descriptive statistics, univariate (unadjusted), and multivariate (adjusted) models in R (version 4.0, Vienna, Austria). See the code here: https://github.com/ucd-brenn/CODEN.

For our regression analyses, we considered various options given our binary outcome variable (being hospitalized or not). In large cohort studies where the outcome variable is common, such as our study, logistic regression models tend to overestimate incident rate ratios [68]. Thus, we evaluated alternatives such as Poisson and negative binomial models, both options in studies with large cohorts and common binary outcome variables [68]. To do so, we first conducted a dispersion test for the outcome variable (hospitalization), showing that the mean (0.29) is slightly larger than the variance (0.20). Poisson distributions describe cases wherein the mean is equal to the variance, whereas, in negative binomial distributions, the variance is larger than the mean. Then, we used the odTest function in the *pscl* package to “to test the null hypothesis that the restriction implicit in the Poisson model is true” ([69], p. 45). This likelihood ratio test resulted in a Chi-Square Test Statistic = -0.286 and p-value = 0.5, lending support for the null hypothesis and suggesting that Poisson regression is preferable. Because our data are under-dispersed relative to the Poisson distribution, we ran Poisson regressions with robust standard errors [68].

We first ran univariate Poisson regressions with robust standard errors to test associations of individual variables with the incident rate ratio (IRR) of hospitalization. We selected variables for multivariate models from relevant literature and display these in Table 1, and we chose to retain independent variables not associated with the dependent variable to account for the possible presence of confounding and suppressor variables [70]. Before running the univariate and multivariate regressions, we standardized the continuous variables listed in Table 1.

In multivariate Poisson regressions with robust standard errors, we evaluated the neighborhood built environment factors associated with the IRR of hospitalization among those who tested positive for SARS-CoV-2, while controlling for individual and neighborhood demographics as well as individual comorbidities. We performed one main analysis for all individuals with complete data, and then two subgroup analyses for non-Hispanic white individuals, and Latinx individuals. We did not compute models for other racial/ethnic groups (e.g., non-Hispanic Black people) given the small size of their subsample in our cohort compared to Latinx and non-Hispanic white people. We were particularly interested in understanding how associations between neighborhood built environment and IRRs of hospitalization varied by race/ethnicity because people of color in the U.S. have higher rates of COVID-19 hospitalization [41] and live in places with disproportionately high exposures to environmental hazards, such as air pollution [71].

To test for potential spatial autocorrelation in the model’s residuals of the Poisson regression with robust standard errors, we computed global Moran’s *Is* for the model residuals. We used the moran.test function in R’s *spdep* package to calculate Moran’s *I* with a distance-based spatial matrix that considers the four closest neighbors to each home address of the patients in our sample with complete records. We created three different spatial matrices for the entire sample and the two subsamples. Moran’s *I* tests were significant for the entire sample, the Latinx subsample, and the non-Hispanic white subsample. Thus, we ran spatial filtering models using the ME function in R’s *spdep* package. Spatial filtering models calculate eigenvectors intended to remove spatial autocorrelations [72]. Through a stepwise process, some eigenvectors are included in the model, and their inclusion reduces the *p*-value of Moran’s *I* of the model’s residual below 0.05.

### 2.5. IRB approval

The Institutional Review Board at The University of Utah and the Colorado Multiple Institutional Review Board (COMIRB) approved the study. We utilized research use agreements for data sharing across the authors’ institutions. COMIRB, which oversees the two hospital systems in this study, waived informed consent for this study due to the retrospective nature of the research: Obtaining informed consent was not feasible and the risk for adverse events was considered extremely low. Names and medical record numbers were removed from the dataset prior to analysis and dates of birth were converted to years of age at the time of the positive test, but other PHI elements that were considered critical to the study were retained. These included the home address and year of testing and hospitalization.

## 3. Results

### 3.1. Descriptive statistics

From May 1 to December 31, 2020, 23,471 adults living in the Denver metropolitan area were diagnosed with SARS-CoV-2 infection through UCH and DH. Of those, we removed 163 records due to incomplete/missing addresses. We imputed missing heights for 159 individuals to calculate BMI. Of the remaining 23,308 records, we removed 4,101 individuals due to missing weight data since BMI could not be calculated. We removed an additional 532 records due to missing race and ethnicity data. Further, we excluded 557 records due to lack of available transit, bike, or walk scores, and 76 records due to lack of available data from the U.S. Census Bureau (e.g., housing or employment data). The final dataset included 18,042 individuals. See S1 Fig in the S1 Appendix for a flowchart describing the selection of patients based on data availability.

Table 2 lists the characteristics of the final cohort. Fig 1 shows the geographic distribution of all cases (1a) and hospitalized cases (1b). Overall, 5,239 individuals (29.03%) were hospitalized within 2 weeks of a positive SARS-CoV-2 test result. Compared to those who were not hospitalized, hospitalized individuals had higher BMI, were older, were more often Latinx or non-Hispanic Black, and had a higher prevalence of the included medical conditions.

**Table 2:** Demographic and clinical characteristics for the final cohort.

	Overall (N = 18,042)	Hospitalized (N = 5,239)	Not Hospitalized (N = 12,803)	p-value
**Age in years (Median, IQR)**	43, 31–57	50, 35–64	41, 30–54	<0.001
Gender (*n*, %)*				<0.001
Women	10,508 (58.2)	2,920 (55.7)	7,588 (59.3)	
Men	7,534 (41.8)	2,318 (44.3)	5,216 (40.7)	
Race/Ethnicity (*n*, %)				<0.001
Non-Hispanic White	8,513 (47.2)	2,011 (38.4)	6,502 (50.8)	
Non-Hispanic Black	1,220 (6.8)	518 (9.9)	702 (5.5)	
NH American Indian/Alaska Native	59 (0.3)	19 (0.4)	40 (0.3)	
Hispanic/Latinx	7,421 (41.1)	2,369 (45.2)	5,052 (39.5)	
Non-Hispanic Asian	618 (3.4)	252 (4.8)	366 (2.9)	
NH Native Hawaiian/Pacific Islander	48 (0.3)	24 (0.5)	24 (0.2)	
NH Mixed Race	163 (0.9)	45 (0.9)	118 (0.9)	
**BMI (Median, IQR)**	29, 25.2–33.8	30, 26.1–35.3	28.6, 24.9–33.3	<0.001
**Tobacco Use (current) (*n*, %)**	2,487 (13.8)	627 (12)	1,860 (14.5)	<0.001
**Medical Conditions (*n*, %)**				
Diabetes	2,054 (11.4)	850 (16.2)	1,204 (9.4)	<0.001
Hypertension	3,427 (19)	1,158 (22.1)	2,269 (17.7)	<0.001
Chronic Kidney Disease	838 (4.6)	390 (7.4)	448 (3.5)	<0.001
Chronic Lung Disease	2,636 (14.6)	1,083 (20.7)	1,553 (12.1)	<0.001

Notes:

* None of the individuals in the cohort had evidence of identifying as transgender, nonbinary, or gender nonconforming in the EHRs. NH = non-Hispanic. Comparisons were calculated using Mann-Whitney U tests (for continuous variables such as age and BMI) and χ ^2^ tests (for categorical variables). To calculate BMI, we imputed height values for 159 people in the cohort (0.88%) for whom height data were not available (see also Section 2.1).

Table 3 summarizes the descriptive statistics for the neighborhood built environments. For example, 24.6% of individuals in the sample live in a multi-family housing unit. Nearly half (47.4%) of the cohort lived within a half-mile of a park, and scores for walkability, bikeability, and transit access varied widely. See S1 and S2 Tables in the S1 Appendix for additional details.

**Table 3:** Descriptive statistics for neighborhood built environment variables for the final cohort (n = 18,042).

Variable	Mean	Median	St. dev.	Min.	Max.
Residential density (households per Km^2^)	889.336	813.54	827.521	0.808	7488.340
Percent overcrowding (ranging between 0 and 1)	0.041	0.023	0.042	0	0.246
Living in a multi-family building (yes = 1, no = 0)	0.246	0	0.354	0	1
Percent multi-family units (ranging between 0 and 1)	0.275	0.218	0.249	0	0.992
PM _2.5_ (μg/m^3^)	8.029	8.140	0.585	5.096	8.902
Proximity to a highway (yes = 1, no = 0)	0.289	0	0.453	0	1
NDVI greenness (number of pixels classified as green in block group)	2484.780	2483	655.591	0	6339
Park access (yes = 1, no = 0)	0.474	0	0.499	0	1
Park acreage (Km^2^)	0.004	0	0.008	0	0.071
Walk Score ^®^ (0–100 index)	42.964	43.656	24.438	0	98.027
Bike Score ^®^ (0–100 index)	57.458	57.583	17.707	1.045	100
Transit Score ^®^ (0–100 index)	34.784	37.671	17.129	0	97.205

### 3.2. Associations between neighborhood built environment and COVID-19 hospitalizations

In univariate analyses, the four domains of neighborhood built environments were significantly associated with the incident rate ratio (IRR) of being hospitalized (Table 4). The IRR was higher for those living in neighborhoods with larger shares of overcrowded households and for those living in a multi-family building (density and crowding). Also, the IRR of hospitalization was higher for people living in neighborhoods with higher PM_2.5_ levels (environmental hazards) and lower for people living in neighborhoods with higher overall greenness, measured through NDVI (environmental amenities). Furthermore, the IRR of hospitalization was higher for people living in neighborhoods with higher Transit Scores^®^.

**Table 4:** Poisson regressions with robust standard errors predicting the incidence rate ratios (IRR) of hospitalization among individuals with positive SARS-CoV-2 PCR tests.

	Univariate models (all cases)			Multivariate model (all cases)		
Variable	IRR	95% CI	p-value	IRR	95% CI	p-value
*Density and crowding*						
Residential density	0.990	0.968–1.012	0.384	0.973	0.938–1.009	0.145
Percent overcrowding	**1.173**	**1.151–1.956**	**<0.001**	1.014	0.983–1.046	0.383
Living in a multi-family building	**1.170**	**1.102–1.242**	**<0.001**	**1.142**	**1.075–1.213**	**<0.001**
Percent multifamily units	1.018	0.996–1.041	0.111	0.975	0.943–1.009	0.151
*Environmental hazards*						
PM_2.5_	**1.244**	**1.210–1.279**	**<0.001**	**1.190**	**1.151–1.230**	**<0.001**
Proximity to a highway	1.031	0.981–1.084	0.226	0.955	0.905–1.007	0.087
*Environmental amenities*						
NDVI	**0.964**	**0.943–0.986**	**0.001**	0.998	0.973–1.022	0.850
Park proximity	1.022	0.977–1.070	0.336	**1.056**	**1.001–1.115**	**0.045**
Park acreage	0.986	0.964–1.009	0.249	0.980	0.952–1.009	0.177
*Mobility*						
Walk Score ^®^	1.004	0.982–1.027	0.713	**0.957**	**0.919–0.996**	**0.033**
Bike Score ^®^	0.984	0.962–1.006	0.150	**0.919**	**0.883–0.956**	**<0.001**
Transit Score ^®^	**1.078**	**1.054–1.102**	**<0.001**	**1.075**	**1.030–1.123**	**<0.001**
*Intercept*				0.274	0.195–0.385	<0.001

Notes: (a) n = 18,042. (b) IRRs in bold are significant at the 0.05 level. (c) The multivariate (adjusted) model controls for age, gender, race/ethnicity, BMI, tobacco smoking, diabetes, hypertension, chronic kidney disease, chronic lung disease, percent housing burdened households, percent essential workers, and percent essential workers commuting via transit. (d) For the multivariate model, Akaike Information Criterion = 21,935. (e) The multivariate model is a spatial filtering model that eliminates spatial autocorrelation. (f) p values and 95% confidence intervals are calculated with robust standard errors. (h) All continuous variables were standardized.

In multivariable analyses adjusting for demographics and comorbidities (age, gender, race/ethnicity, BMI, tobacco smoking, diabetes, hypertension, chronic kidney disease, chronic lung disease), certain elements of the neighborhood built environment remained significantly associated with the IRR of hospitalization (Table 4). The IRR was higher for individuals living in an apartment (*p* < 0.001), but not for the other density and crowding variables (e.g., percent overcrowding). Specifically, the IRR of hospitalization was 14.2% higher for people living in a multi-family building than for people in a single-family unit. Living in a neighborhood with higher PM_2.5_ levels was associated with a higher IRR of hospitalization (*p* < 0.001). None of the environmental amenities (parks and NDVI) showed significant protective associations, but living within a half-mile of a park was linked with a higher IRR of hospitalization, although this result was significant only at the 0.05 level. Results for mobility were mixed. The IRR of hospitalization was higher for individuals whose neighborhood had a lower Walk Score ^®^ (*p* < 0.05), lower Bike Score ^®^ (*p* < 0.001), and higher Transit Score ^®^ (*p* < 0.001). Incidence rate ratios (IRRs) and significant levels for the control variables are in S3 Table in S1 Appendix.

Tukey-adjusted post-hoc pairwise comparisons showed that, when controlling for other variables, non-Hispanic white people with SARS-CoV-2 were less likely to be hospitalized than people of color (see S4 Table in the S1 Appendix). Also, to test whether adding the built environment variables to the individual-level comorbidities and demographics improved the model fit, we ran a Poisson regression with robust standard errors that only included the control variables. The model with the built environment variables had a lower Akaike Information Criterion (AIC = 21,934.92) and a lower Bayesian Information (BIC = 22,761.77) than the model without such variables (AIC = 21,952.56, BIC = 22857.41). This shows that adding the built environment variables to the individual-level comorbidities and demographics improved the model fit, albeit only slightly.

In subgroup models of non-Hispanic white and Latinx subsamples (Table 5 and S5 Table in the S1 Appendix), we observed some variations in the significant associations and effect sizes. Higher PM_2.5_ levels had stronger associations with hospitalization in the Latinx subsample (IRR = 1.347, 95% CI = 1.289–1.407) than in the non-Hispanic white subsample (IRR = 1.087, 95% CI = 1.041–1.136), and the two confidence intervals did not overlap. Also, a lower Walk Score^®^ was associated with a higher incidence rate ratio (IRR) of hospitalization for Latinx individuals, but not for non-Hispanic white individuals. On the contrary, a lower Bike Score^®^, higher Transit Score^®^, higher percent of overcrowded units, and living within > ½ mile from a park (within 800 m) were all associated with a higher IRR of hospitalization in the non-Hispanic white sample, but not in the Latinx sample. Finally, living in a multi-family unit was associated with a higher IRR of hospitalization in both samples, but the effect size was larger in the non-Hispanic white cohort.

**Table 5:** Poisson regressions with robust standard errors predicting the incidence rate ratios (IRR) of hospitalizations for individuals who tested positive for SARS-CoV-2, broken down between two samples: Non-Hispanic white and Latinx people.

	Non-Hispanic white (n = 8,513)			Latinx (n = 7,421)		
Variable	IRR	95% CI	p-value	IRR	95% CI	p-value
*Density and crowding*						
Residential density	0.956	0.905–1.010	0.109	0.985	0.933–1.040	0.589
Percent overcrowding	**1.064**	**1.004–1.129**	**0.036**	1.005	0.948–1.065	0.878
Living in a multi-family building	**1.265**	**1.143–1.401**	**<0.001**	**1.126**	**1.017–1.247**	**0.022**
Percent multifamily units	0.978	0.926–1.032	0.414	1.010	0.957–1.066	0.719
*Environmental hazards*						
PM_2.5_	**1.087**	**1.041–1.136**	**<0.001**	**1.347**	**1.289–1.407**	**<0.001**
Proximity to a highway	1.009	0.921–1.105	0.844	1.025	0.936–1.122	0.601
*Environmental amenities*						
NDVI	1.013	0.978–1.049	0.474	0.968	0.934–1.002	0.065
Park proximity	**1.144**	**1.046–1.251**	**0.003**	0.964	0.882–1.054	0.425
Park acreage	0.958	0.917–1.002	0.06	1.005	0.962–1.051	0.817
*Mobility*						
Walk Score ^®^	1.007	0.940–1.078	0.848	**0.874**	**0.816–0.935**	**<0.001**
Bike Score ^®^	**0.915**	**0.860–0.974**	**0.005**	0.971	0.913–1.034	0.362
Transit Score ^®^	**1.112**	**1.041–1.187**	**0.002**	1.018	0.953–1.087	0.604
*Intercept*	0.196	0.180–0.213	<0.001	0.235	0.216–0.256	<0.001

Notes: (a) IRRs in bold are significant at the 0.05 level. (b) The multivariate models control for age, gender, BMI, tobacco smoking, diabetes, hypertension, chronic kidney disease, chronic lung disease, percent housing burdened households, percent essential workers, and percent essential workers commuting via transit. (c) Non-Hispanic white model, Akaike Information Criterion = 9385.9. Latinx model, Akaike Information Criterion = 9453.6. (d) These models are spatial filtering models that eliminate spatial autocorrelation. (e) p-values and 95% confidence intervals are calculated with robust standard errors.

## 4. Discussion

### 4.1. Summary of findings

Among a cohort of more than 18,000 individuals with SARS-CoV-2 infection, living in a multi-family building, living in a neighborhood with higher PM_2.5_ levels, and living in a neighborhood with lower walkability and bikeability were associated with a greater incident rate ratio (IRR) of hospitalization, even when controlling for socioeconomic vulnerability and individual-level demographic and medical characteristics. NDVI was associated with a lower IRR of hospitalization in univariate models but the association did not remain significant in multivariable models. Also, in multivariate models, living within a half-mile of a park was associated with a higher IRR of hospitalizations. And although walkability and bikeability were observed to be protective against hospitalization, transit score–a marker of transit quality and access–was associated with a higher IRR of hospitalization.

When stratifying by race and ethnicity, we observed notable variations in the results for the Latinx and non-Hispanic white cohorts. For example, although higher PM_2.5_ was significantly associated with a higher IRR of hospitalizations in both cohorts, the effect size was much larger for the Latinx than the non-Hispanic white cohort. This suggests that reducing harmful emissions might provide greater benefits to Latinx populations, a group that was hospitalized at 2.8 times the rate of non-Hispanic white populations in the U.S. during the first year of the COVID-19 pandemic [26]. In contrast to PM_2.5_ levels, markers of density and overcrowding were more strongly associated with a greater IRR of hospitalization among non-Hispanic white than Latinx individuals with SARS-CoV-2 infection, when controlling for other factors as described above. Similarly, living within a half-mile of a park was associated with a higher IRR of hospitalization among non-Hispanic white people but not among Latinx people. The reasons for the differences in these associations between the two cohorts are unclear.

Of the environmental hazards we studied, higher PM_2.5_ levels were most consistently associated with a greater IRR of hospitalization. This finding supports the results of previous work showing that long-term exposure to air pollution–especially PM_2.5_ –is associated with more SARS-CoV-2 transmission, more severe COVID-19, and higher COVID-19 mortality [13,15,73]. Given the well-established link between pollution and cardiovascular disease [74], a known risk factor for severe COVID-19 [7], it is possible that undiagnosed cardiovascular disease or unrecognized cardiovascular damage associated with higher PM_2.5_ levels drives poorer outcomes among those who become ill with COVID-19. Long-term PM_2.5_ exposure can also lead to chronic respiratory stress, which in turn can render individuals susceptible to complications from COVID-19 [75]. A prior study found that individuals with chronic lung disease who were exposed to higher levels were PM_2.5_ were significantly more likely to be hospitalized from COVID-19, suggesting an exacerbation of the underlying disease [76]. PM_2.5_ and other forms of air pollution may also worsen disease severity by increasing epithelial permeability, increasing expression of ACE2 receptors in the airways, and causing oxidative stress, enhanced inflammatory responses, and immune dysregulation [77–79]. Most of these studies have relied on ecological data, which, although informative, have several limitations. Only a handful of studies in the US have relied on individual-level patient data [16,76]. One found that the association between PM_2.5_ and COVID-19 hospitalizations was contingent on patients having pre-existing asthma or chronic pulmonary disorder [76]. Similar to our results, Bowe and colleagues found that the annual average PM_2.5_ levels in 2018 were linked with an increased risk of hospitalization among a very large cohort of United States Veterans and that Black individuals were more susceptible to the effect of PM_2.5_ on COVID-19 illness than white individuals [16].

Our study adds strong evidence of associations between air pollution and COVID-19 severity, particularly among Latinx populations. These findings are particularly important given that people of color and socioeconomically disadvantaged groups in the United States are systematically exposed to higher air pollution levels [71,80], and that there exist substantial disparities in the risk of COVID-19 infection by race/ethnicity and socioeconomic conditions [81]. The confluence of environmental injustices with infection rate disparities and disease severity among people of color illustrates the compound effects of these forces on health outcomes.

Our findings on mobility partially align with those of previous work [20,82,83] showing that living in more walkable and bikeable neighborhoods lowers the IRR of hospitalization among people with COVID-19. A recent study by Sallis and colleagues showed that being physically inactive was linked to higher risks of hospitalization, admission to an Intensive Care Unit, and death [84]. Thus, we hypothesize that living in places conducive to walking and/or biking leads to more physical activity, which may be protective against those outcomes. For unclear reasons, the effects of bikeability and walkability varied significantly between the two sub-cohorts, suggesting the need for further research into whether these scores may reflect different experiences for racial and ethnic subgroups in the Denver metropolitan area and elsewhere.

We noted a harmful association between better transit access and hospitalization risk. Wang and colleagues theorized that transit riders might have a higher risk of infection due to possible prolonged exposure to SARS-CoV-2 in small indoor settings [20]. Although this theory would not fully explain our findings on the risk for hospitalization among those who contracted SARS-CoV-2, it is possible that transit riding may have other unidentified health risks or that people who continued to use public transit during the first year of the COVID-19 pandemic may have had health conditions not captured in our study [85]. Like the bike and walk score results, associations with transit scores varied between the subgroups, limiting the generalizability of the finding at this time.

We also found that living within a half-mile of a park was associated with a higher IRR of hospitalization for people with SARS-CoV-2 infection in the entire cohort and the non-Hispanic white subsample, but not the Latinx subsample. These findings are unlike those of previous studies, which largely found that higher densities of green space were associated with

COVID-19-related mortality and hospitalization risks [16–21]. The reasons for these differences are unclear, but they might be due to our inclusion of many other built environment variables in our model, including markers of environmental hazards, mobility, and density and crowding, which other studies on the association between green space and COVID-19 outcomes rarely considered.

### 4.2. Strengths, limitations, and future research

Our study has several strengths. Importantly, we used individual-level demographic and clinical data for >18,000 individuals with SARS-CoV-2 infection and linked each case to secondary data sources, using the geocoded addresses to measure neighborhood built environment domains and additional control variables describing socioeconomic factors. To our knowledge, this is one of the first and largest studies to utilize individual-level data to determine environmental correlates of COVID-19 disease severity. Further, we considered how associations between neighborhood built environment and COVID-19 hospitalization differed between Latinx and non-Hispanic white people, and found notable differences in such associations, such as the larger impact of particulate matter in the Latinx cohort.

Our study also has several limitations. First, we used an observational design, which may be subject to residual confounding due to unmeasured covariates or missing data. We tried to mitigate this concern by including a wide range of individual-level covariates but did not have individual-level data describing socioeconomic status, and therefore we used such data at the census block group level. Certain comorbid conditions may not have been documented in the EHR leading to possible misclassification. In particular, chronic lung disease encompasses a range of conditions with potentially differing susceptibilities to complications resulting from environmental exposures such as particulate matter. Second, the large number of records censored from the cohort due to missing weight/BMI values or mobility score values (e.g., Transit Score ^®^) may have skewed the cohort toward those with more healthcare utilization due to higher healthcare needs. Third, the reason for hospitalization was not available in the dataset, and therefore some patients might have incidentally been positive for SARS-CoV-2 upon screening but were not hospitalized *because of* COVID-19. The Infection Prevention teams at DH and UCH report that asymptomatic SARS-CoV-2 infections typically represented less than 5% of hospitalized cases in 2020, but the exact prevalence of this scenario in our cohort is unknown.

Fourth, we relied on PM_2.5_ concentrations for the year 2016. It is possible that in response to lockdown policies during the pandemic, fluctuations and short-term PM_2.5_ concentrations may have differed from 2016 levels. Fifth, and relatedly, some of the data sources we used do not completely align temporally; for example, demographic data are for 2015–2019, hospitalization data are for 2020, and data about walkability, bikeability, and transit access are for 2021. Sixth, we did not include measures of park quality because data needed to model quality were not available homogeneously across the various jurisdictions. Seventh, the results of the two subgroup analyses for non-Hispanic white and Latinx individuals might include inflated false positive rates [86]. Yet many statistically significant associations for key variables (e.g., PM _2.5_) in the two subsamples were at the 0.01 levels or lower, which strengthens our confidence in the accuracy of the results. Finally, our dataset included all SARS-CoV-2-positive results from the two largest public hospitals in the Denver metropolitan area and thus accounted for many, but not all, of the region’s cases. It is possible that individuals who tested for SARS-CoV-2 outside of these healthcare systems may be healthier and differently impacted by the environmental variables assessed in this study.

Future research could build on our line of inquiry in several ways. Reproducing these results in other jurisdictions will be critical to understanding the generalizability and validity of our findings, particularly in jurisdictions with different racial and ethnic distributions. Further, environmental exposures describing only one’s neighborhood fail to represent other places where individuals spend significant time, an issue defined as the neighborhood effect averaging problem (NEAP) [87]. Therefore, subsequent studies are also needed to examine the effects of environmental exposures in the locations where one works or partakes in leisure. Finally, future studies could assess the effects of different components of air pollution, including different particulate matter sizes.

### 4.3. Implications for urban planning, urban policy, and public health

The findings of this study show that certain neighborhood built environment characteristics are associated with increased or decreased IRR of being hospitalized among individuals who tested positive for SARS-CoV-2. These associations are statistically significant even when controlling for several demographic characteristics and comorbidities. As such, these findings could be used to inform future public health and urban planning interventions that could help limit the severity of COVID-19 and other airborne infectious diseases.

Our results for density and crowding could help direct public health vaccination and testing efforts to areas with a higher prevalence of multifamily housing. These efforts could mitigate the risk of severe disease through the prevention of infection and augmented immunity from vaccines, and by encouraging earlier access to therapeutics for those who test positive for SARS-CoV-2 [88–90]. Living in a transit-rich neighborhood was also associated with a higher risk for COVID-19 hospitalization, so public health efforts targeting these neighborhoods could be helpful, as could additional public health measures like masking and educational messaging on transit itself.

Ambient PM_2.5_ exposure is an increasing concern worldwide due to its association with numerous poor health outcomes [91–93] and its disproportionate effect on people of color [94–96]. Our findings underscore the urgency of lowering PM emissions to improve public health, especially respiratory-related diseases. Ways to reduce PM emissions include facilitating opportunities for public transport or active transportation, controlling industrial sources, limiting highway expansions, and increasing green space [97]. To have the greatest impact, principles of environmental justice should be at the core of future planning efforts.

Our findings for mobility suggest the need to make existing neighborhoods more walkable and bikeable, and to build new neighborhoods conducive to walking and biking, investing in pedestrian and cycling infrastructure and promoting mixed land uses and residential densities. Since walkability was particularly protective for Latinx individuals, planners should prioritize pedestrian investments in majority-Latinx neighborhoods. Walkability investments in communities of color should follow a holistic environmental justice approach with meaningful community engagement in planning [98]. In this context, investments need to ensure that increased walkability and bikeability do not result in the over-policing of people of color, as research has shown disproportionate cycling citations in Black and Latinx communities [99].

## 5. Conclusion

This study contributes to the literature on the associations between the built environment and COVID-19 outcomes by using a holistic definition of neighborhood built environment, leveraging a large cohort with individual-level demographic and comorbidity data, and focusing on hospitalizations (a marker of severity). Of more than 18,000 individuals with SARS-CoV-2 in our cohort, those who lived in multi-family housing units and those exposed to higher levels of PM_2.5_ were at higher risk of hospitalization, even when controlling for established risk factors such as age, weight, and medical conditions. Higher walkability, higher bikeability, and lower transit access were associated with a lower IRR of hospitalization. Results for sub-samples of Latinx and non-Hispanic white individuals showed significant variations, including higher PM_2.5_ levels being particularly harmful to Latinx individuals.

Many of the neighborhood characteristics associated with lower COVID-19 hospitalizations in our study–more walkability and bikeability and less pollution–are considered best practices in urban planning to improve public health, boost livability, and address climate change [100,101]. By heeding lessons learned from COVID-19, we may see public health and environmental benefits that extend well beyond the improved control of future respiratory pandemics.

## Supporting information

S1 Appendix (DOCX)
