# Supplementary material for: The neighborhood built environment and COVID-19 hospitalizations
Source: PLoS One. 2023 Jun 14;18(6):e0286119. doi: 10.1371/journal.pone.0286119 (PMC10266617; doi:10.1371/journal.pone.0286119)
Supplement: S1 Appendix — (DOCX) [file pone.0286119.s001.docx]

**The neighborhood built environment and COVID-19 hospitalizations**

S1 Appendix


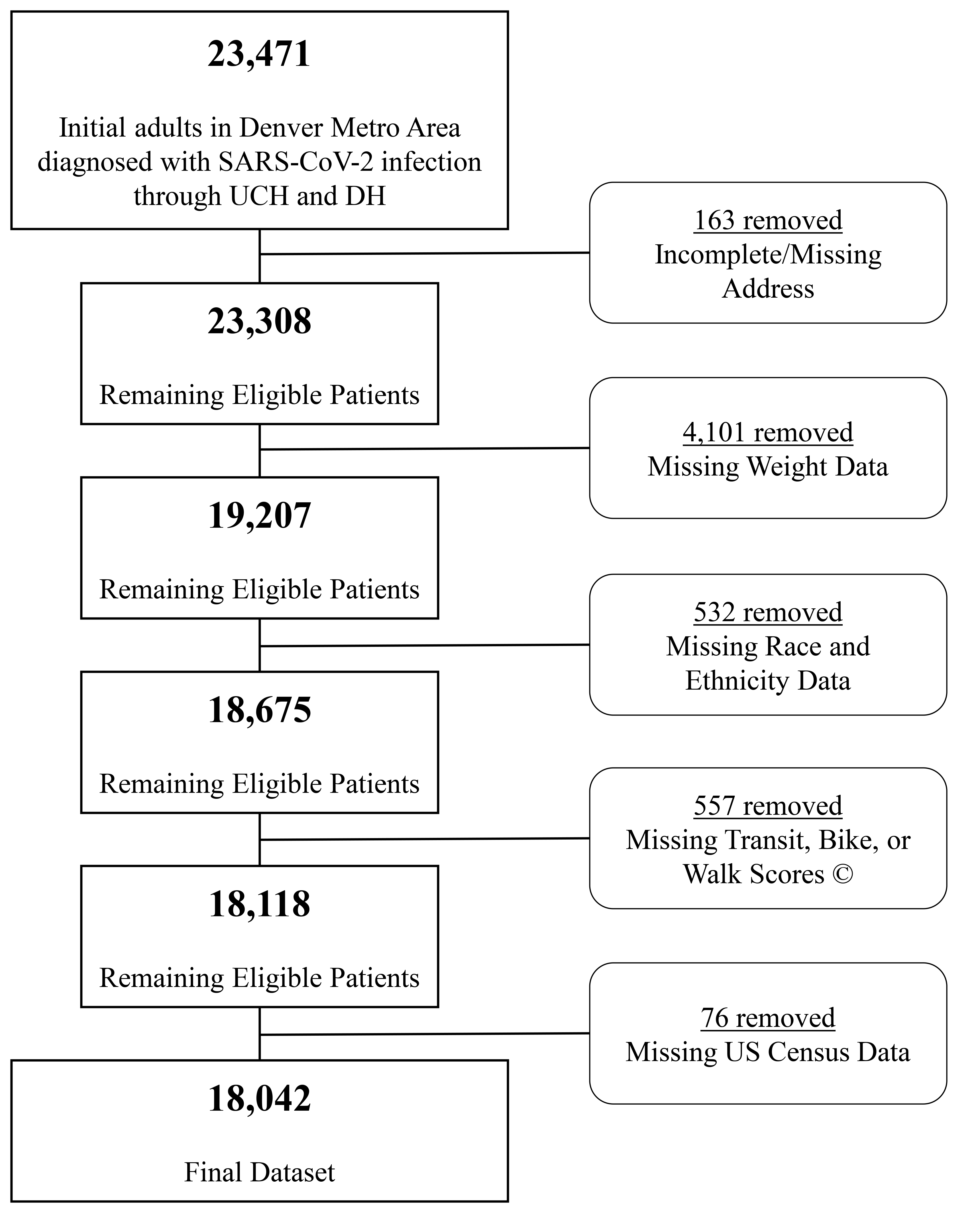


Fig S1. Flowchart describing the selection of patients based on data availability.

Table S1. Descriptive statistics for the numeric variables

| **Variable** | **Mean** | **St. Dev.** | **Min.** | **25%** | **50%** | **75%** | **Max.** |
| --- | --- | --- | --- | --- | --- | --- | --- |
| Hospitalization | 0.290 | 0.454 | 0 | 0 | 0 | 1 | 1 |
| Age | 45.047 | 17.088 | 18 | 31 | 43 | 57 | 102 |
| Body Mass Index (BMI) | 30.209 | 7.183 | 11.90 | 25.20 | 29.02 | 33.80 | 86.78 |
| Currently smokes tobacco | 0.138 | 0.345 | 0 | 0 | 0 | 0 | 1 |
| Diabetes | 0.113 | 0.318 | 0 | 0 | 0 | 0 | 1 |
| Hypertension | 0.190 | 0.392 | 0 | 0 | 0 | 0 | 1 |
| Chronic kidney disease | 0.046 | 0.210 | 0 | 0 | 0 | 0 | 1 |
| Chronic lung disease | 0.146 | 0.353 | 0 | 0 | 0 | 0 | 1 |
| Percent housing burdened households | 0.359 | 0.264 | 0 | 0.264 | 0.382 | 0.464 | 0.762 |
| Percent essential workers | 0.721 | 0.094 | 0.447 | 0.669 | 0.741 | 0.791 | 0.912 |
| Percent essential workers commuting via transit | 0.032 | 0.028 | 0 | 0.013 | 0.025 | 0.043 | 0.217 |
| Residential density | 889.336 | 827.521 | 0.808 | 430.052 | 813.541 | 1079.724 | 7488.339 |
| Percent overcrowding | 0.041 | 0.042 | 0 | 0.0108 | 0.023 | 0.061 | 0.246 |
| Living in a multi-family building | 0.246 | 0.354 | 0 | 0 | 0 | 0 | 1 |
| Percent multi-family units | 0.275 | 0.249 | 0 | 0.058 | 0.218 | 0.411 | 0.992 |
| PM 2.5 | 8.029 | 0.585 | 5.096 | 7.691 | 8.140 | 8.485 | 8.902 |
| Proximity to a highway | 0.289 | 0.453 | 0 | 0 | 0 | 1 | 1 |
| NDVI greenness | 2448.780 | 655.591 | 0 | 2150 | 2483 | 2880 | 6339 |
| Park access | 0.474 | 0.499 | 0 | 0 | 0 | 1 | 1 |
| Park acreage | 0.004 | 0.008 | 0 | 0 | 0 | 0.006 | 0.071 |
| Walk Score | 42.964 | 24.438 | 0 | 23.219 | 43.656 | 62.278 | 98.027 |
| Bike Score | 57.458 | 17.707 | 1.045 | 46.616 | 57.584 | 69.477 | 100 |
| Transit Score | 34.784 | 17.129 | 0 | 25.204 | 37.671 | 44.485 | 97.205 |

Note: n = 18,042

Table S2. Percentages for the categorical variables

| **Gender** |  |
| --- | --- |
| Female | 58.241% |
| Male | 41.759% |
| **Race and ethnicity** |  |
| Latinx | 41.132% |
| Non-Hispanic Black | 6.762% |
| Non-Hispanic White | 47.184% |
| Non-Hispanic Asian | 3.425% |
| Non-Hispanic Native American or Alaskan Native | 0.327% |
| Non-Hispanic Native Hawaiian or Other Pacific Islander | 0.266% |
| Non-Hispanic Mixed Race | 0.904% |

Note: n = 18,042

Table S3. Incidence rate ratios (IRR) of hospitalization for the entire sample

|  | **Univariate models** | | | **Multivariate model** | | |
| --- | --- | --- | --- | --- | --- | --- |
|  | IRR | 95% CI | p-value | IRR | 95% CI | p-value |
| *Density and crowding* |  |  |  |  |  |  |
| Residential density | 0.990 | 0.968-1.012 | 0.384 | 0.973 | 0.938-1.009 | 0.145 |
| Percent overcrowding | **1.173** | **1.151-1.956** | **<0.001** | 1.014 | 0.983-1.046 | 0.383 |
| Living in a multi-family building | **1.170** | **1.102-1.242** | **<0.001** | **1.142** | **1.075-1.213** | **<0.001** |
| Percent multi-family units | 1.018 | 0.996-1.041 | 0.111 | 0.975 | 0.943-1.009 | 0.151 |
| *Environmental hazards* |  |  |  |  |  |  |
| PM _2.5_ | **1.244** | **1.210-1.279** | **<0.001** | **1.190** | **1.151-1.230** | **<0.001** |
| Proximity to a highway | 1.031 | 0.981-1.084 | 0.226 | 0.955 | 0.905-1.007 | 0.087 |
| *Environmental amenities* |  |  |  |  |  |  |
| NDVI greenness | **0.964** | **0.943-0.986** | **0.001** | 0.998 | 0.973-1.022 | 0.850 |
| Park access | 1.022 | 0.977-1.070 | 0.336 | **1.056** | **1.001-1.115** | **0.045** |
| Park acreage | 0.986 | 0.964-1.009 | 0.249 | 0.980 | 0.952-1.009 | 0.177 |
| *Mobility* |  |  |  |  |  |  |
| Walk Score ^®^ | 1.004 | 0.982-1.027 | 0.713 | **0.957** | **0.919-0.996** | **0.033** |
| Bike Score ^®^ | 0.984 | 0.962-1.006 | 0.150 | **0.919** | **0.883-0.956** | **<0.001** |
| Transit Score ^®^ | **1.078** | **1.054-1.102** | **<0.001** | **1.075** | **1.030-1.123** | **<0.001** |
|  |  |  |  |  |  |  |
| *Individual-level control variables* |  |  |  |  |  |  |
| Age | **1.338** | **1.310-1.366** | **<0.001** | **1.380** | **1.349-1.412** | **<0.001** |
| Gender (male) | **1.107** | **1.057-1.159** | **<0.001** | **1.106** | **1.060-1.155** | **<0.001** |
| Race: Non-Hispanic Black* | 1.318 | 0.905-1.920 | 0.149 | 1.056 | 0.750-1.486 | 0.755 |
| Race: Non-Hispanic White* | 0.733 | 0.505-1.064 | 0.103 | 0.740 | 0.528-1.038 | 0.082 |
| Race: Non-Hispanic Asian* | 1.266 | 0.864-1.856 | 0.226 | 1.153 | 0.815-1.632 | 0.420 |
| Race: Latinx* | 0.991 | 0.683-1.438 | 0.963 | 0.916 | 0.654-1.283 | 0.609 |
| Race: Non-Hispanic Native Hawaiian or Other Pacific Islander* | 1.553 | 0.974-2.474 | 0.064 | 1.264 | 0.819-1.949 | 0.290 |
| Race: Non-Hispanic Mixed Race* | 0.857 | 0.549-1.339 | 0.498 | 0.829 | 0.549-1.251 | 0.371 |
| Body Mass Index (BMI) | **1.160** | **1.138-1.182** | **<0.001** | **1.118** | **1.096-1.141** | **<0.001** |
| Currently smokes tobacco | **0.850** | **0.791-0.914** | **<0.001** | **0.815** | **0.761-0.873** | **<0.001** |
| Diabetes | **1.508** | **1.424-1.597** | **<0.001** | 1.060 | 0.995-1.130 | 0.071 |
| Hypertension | **1.210** | **1.147-1.277** | **<0.001** | **0.780** | **0.735-0.829** | **<0.001** |
| Chronic kidney disease | **1.652** | **1.530-1.783** | **<0.001** | **1.230** | **1.132-1.336** | **<0.001** |
| Chronic lung disease | **1.523** | **1.445-1.606** | **<0.001** | **1.413** | **1.341-1.489** | **<0.001** |
| *Neighborhood-level control variables* |  |  |  |  |  |  |
| Percent housing burdened households | **1.123** | **1.096-1.149** | **<0.001** | **1.037** | **1.006-1.070** | **0.019** |
| Percent essential workers | **0.865** | **0.847-0.883** | **<0.001** | 0.974 | 0.942-1.008 | 0.139 |
| Percent essential workers commuting via transit | 1.016 | 0.994-1.039 | 0.145 | 0.986 | 0.958-1.015 | 0.333 |
| Intercept |  |  |  | 0.274 | 0.195-0.385 | <0.001 |
| *Akaike Information Criterion* |  |  |  | 21,935 | | |

Notes: (a) n = 18,042. (b) * For race/ethnicity, the reference group is Non-Hispanic Native American/Alaskan Native. (c) p values and 95% confidence intervals are calculated with robust standard errors. (d) The multivariate (adjusted) model is a spatial filtering model that eliminates spatial autocorrelation. (e) p values and 95% confidence intervals are calculated with robust standard errors in a Poisson regression. (f) All continuous variables were standardized.

Table S4. Tukey-adjusted pairwise comparisons in the incidence rate ratios (IRRs) of hospitalization between racial/ethnic groups (multivariate model)

| **Estimated marginal means (incidence rate ratios)** | | | |  |
| --- | --- | --- | --- | --- |
| NH Black | 0.386 |  |  | |
| NH White | 0.255 |  |  | |
| NH Asian | 0.42 |  |  | |
| Latinx | 0.314 |  |  | |
| NH Native Hawaiian or Other Pacific Islander | 0.455 |  |  | |
| NH Mixed Race | 0.312 |  |  | |
| NH Native American/Alaskan Native | 0.311 |  |  | |

| **Pairwise comparisons** | | | |  |
| --- | --- | --- | --- | --- |
|  | **Estimated difference (incidence rate ratios)** | **z ratio** | **p-value** | |
| NH Native American/Alaskan Native - NH Asian | 0.74 | -1.264 | 0.8684 | |
| NH Native American/Alaskan Native - NH Black | 0.807 | -0.918 | 0.9699 | |
| NH Native American/Alaskan Native - Latinx | 0.989 | -0.049 | 1 | |
| NH Native American/Alaskan Native - NH Mixed Race | 0.996 | -0.014 | 1 | |
| NH Native American/Alaskan Native - NH Native Hawaiian or Other Pacific Islander | 0.684 | -1.236 | 0.8804 | |
| NH Native American/Alaskan Native - NH White | 1.217 | 0.849 | 0.9797 | |
| NH Asian - NH Black | 1.091 | 1.114 | 0.9239 | |
| **NH Asian - Latinx** | **1.337** | **4.278** | **<0.001** | |
| NH Asian - Mixed | 1.347 | 1.833 | 0.5256 | |
| NH Asian - NH Native Hawaiian or Other Pacific Islander | 0.925 | -0.366 | 0.9998 | |
| **NH Asian - NH White** | **1.645** | **7.328** | **<0.001** | |
| **NH Black - Latinx** | **1.226** | **4.077** | **<0.001** | |
| NH Black - NH Mixed Race | 1.235 | 1.353 | 0.8265 | |
| NH Black - NH Native Hawaiian or Other Pacific Islander | 0.848 | -0.79 | 0.986 | |
| **NH Black - NH White** | **1.509** | **7.856** | **<0.001** | |
| Latinx - NH Mixed Race | 1.008 | 0.05 | 1 | |
| Latinx - NH Native Hawaiian or Other Pacific Islander | 0.692 | -1.792 | 0.5538 | |
| **Latinx - NH White** | **1.231** | **5.816** | **<0.001** | |
| NH Mixed Race - NH Native Hawaiian or Other Pacific Islander | 0.686 | -1.486 | 0.7536 | |
| NH Mixed Race - NH White | 1.222 | 1.322 | 0.8417 | |
| NH Native Hawaiian or Other Pacific Islander - NH White | 1.78 | 2.793 | 0.077 | |

Notes: a. Number of observations = 18,042. b. NH = Non-Hispanic. c. Estimated marginal means represent estimated values of the incidence rate ratios of hospitalization based on the variables regression represented in Table S3 (i.e., controlling for several variables). d. Incidence rate ratios in bold are significant at the 0.05 level.

Table S5. Incidence rate ratios (IRRs) of hospitalization for the two sub-samples (multivariate models)

|  | **Non-Hispanic white** (n = 8,513) | | | **Latinx** (n = 7,421) | | |
| --- | --- | --- | --- | --- | --- | --- |
|  | IRRs | 95% CI | p-value | IRRs | 95% CI | p-value |
| *Density and crowding* |  |  |  |  |  |  |
| Residential density | 0.956 | 0.905-1.010 | 0.109 | 0.985 | 0.933-1.040 | 0.589 |
| Percent overcrowding | **1.064** | **1.004-1.129** | **0.036** | 1.005 | 0.948-1.065 | 0.878 |
| Living in a multi-family building | **1.265** | **1.143-1.401** | **<0.001** | **1.126** | **1.017-1.247** | **0.022** |
| Percent multifamily units | 0.978 | 0.926-1.032 | 0.414 | 1.010 | 0.957-1.066 | 0.719 |
| *Environmental hazards* |  |  |  |  |  |  |
| PM_2.5_ | **1.087** | **1.041-1.136** | **<0.001** | **1.347** | **1.289-1.407** | **<0.001** |
| Proximity to a highway | 1.009 | 0.921-1.105 | 0.844 | 1.025 | 0.936-1.122 | 0.601 |
| *Environmental amenities* |  |  |  |  |  |  |
| NDVI | 1.013 | 0.978-1.049 | 0.474 | 0.968 | 0.934-1.002 | 0.065 |
| Park proximity | **1.144** | **1.046-1.251** | **0.003** | 0.964 | 0.882-1.054 | 0.425 |
| Park acreage | 0.958 | 0.917-1.002 | 0.06 | 1.005 | 0.962-1.051 | 0.817 |
| *Mobility* |  |  |  |  |  |  |
| Walk Score ^®^ | 1.007 | 0.940-1.078 | 0.848 | **0.874** | **0.816-0.935** | **<0.001** |
| Bike Score ^®^ | **0.915** | **0.860-0.974** | **0.005** | 0.971 | 0.913-1.034 | 0.362 |
| Transit Score ^®^ | **1.112** | **1.041-1.187** | **0.002** | 1.018 | 0.953-1.087 | 0.604 |
| *Individual-level control variables* |  |  |  |  |  |  |
| Age | **1.405** | **1.352-1.461** | **<0.001** | **1.360** | **1.309-1.414** | **<0.001** |
| Gender (male) | 1.055 | 0.980-1.136 | 0.158 | **1.215** | **1.128-1.308** | **<0.001** |
| Body Mass Index (BMI) | **1.141** | **1.102-1.181** | **<0.001** | **1.103** | **1.066-1.142** | **<0.001** |
| Currently smokes tobacco | 0.950 | 0.844-1.070 | 0.400 | **0.731** | **0.650-0.823** | **<0.001** |
| Diabetes | 1.051 | 0.920-1.201 | 0.462 | 1.103 | 0.966-1.260 | 0.148 |
| Hypertension | **0.870** | **0.788-0.960** | **0.006** | **0.737** | **0.668-0.814** | **<0.001** |
| Chronic kidney disease | **1.157** | **1.007-1.329** | **0.040** | **1.353** | **1.177-1.554** | **<0.001** |
| Chronic lung disease | **1.236** | **1.124-1.360** | **<0.001** | **1.690** | **1.536-1.858** | **<0.001** |
| *Neighborhood-level control variables* |  |  |  |  |  |  |
| Percent housing burdened households | 1.010 | 0.968-1.054 | 0.659 | 1.017 | 0.975-1.061 | 0.439 |
| Percent essential workers | 0.943 | 0.887-1.004 | 0.067 | 0.999 | 0.939-1.063 | 0.970 |
| Percent essential workers commuting via transit | 0.995 | 0.947-1.044 | 0.827 | 1.007 | 0.960-1.058 | 0.768 |
| Intercept | 0.196 | 0.180-0.213 | <0.001 | 0.235 | 0.216-0.256 | <0.001 |
| *Akaike Information Criterion* |  |  | 9385.9 |  |  | 9453.6 |

Notes: (a) Incidence rate ratios in bold are significant at the 0.05 level. (b) These models are spatial filtering models that eliminate spatial autocorrelation. (c) p values and 95% confidence intervals are calculated with robust standard errors in a Poisson regression. (d) All continuous variables were standardized.
